# Unusual Finding in a Patient Presenting with Abdominal Pain and Dyspnea

**DOI:** 10.5334/jbsr.3549

**Published:** 2024-05-03

**Authors:** Wisam Al-Saad, Nasroola Damry, Gerogiana Pintea Bentea

**Affiliations:** 1 Department of Radiology, CHU Brugmann, Brussels Belgium; 2 Department of Radiology, CHU Brugmann, Brussels Belgium; 3 Department of Cardiology, CHU Brugmann, Brussels Belgium

**Keywords:** cardiac angiosarcoma, clinical presentation, diagnostic imaging

## Abstract

This article discusses the rarity of primary cardiac tumors, particularly angiosarcomas, accounting for 10% of primary cardiac neoplasms. The article highlights the challenge of early diagnosis due to nonspecific symptoms. The importance of sectional imaging, particularly computed tomography (CT) scans, in emergent situations is emphasized.

*Teaching point:* Despite the rarity of primary cardiac tumors, radiologists should remain alert, especially when pericardial effusion is present.

## Introduction

Cardiac tumors are rare and can be categorized as either primary or metastatic. Sarcoma is a rare malignant primary cardiac tumor that accounts for 10% of all primary cardiac neoplasms. Cardiac angiosarcoma is characterized by its aggressive local growth and often leading to early metastasis [1, 2].

Currently, there is no universally standardized treatment protocol. Radical resection stands as the foremost efficacious treatment [3].

We report an unusual finding of primary cardiac angiosarcoma.

## Case Report

A 45-year-old female patient presented to the emergency department for abdominal bloating for 3 days with diffuse pain but more severe in the epigastrium. She reported experiencing intermittent abdominal bloating for 1 year, alongside a recent escalation in epigastric pain over the course of the last month.

A computed tomography (CT) scan was conducted, revealing a moderate quantity of ascites and, additionally, a significant pericardial effusion.

Evidence of cardiac tamponade necessitated urgent drainage, resulting in the removal of a total of 1,150 mL of hemorrhagic fluid.

**Figure 1:**
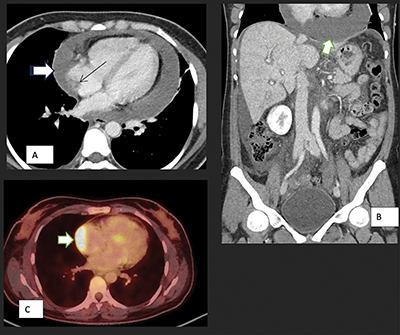
Axial **(A)** and coronal **(B)** CT scan images show pericardial effusion (white arrow) and enhancing pericardium, evidence of right heart failure with IVC enlargement and periportal oedema, features of early cardiac tamponade. The thin arrow points to the soft tissue mass in the atrium. Axial PET-CT **(C)** shows pathological metabolic activity in the known mass in the right atrium (arrow) extending along the pericardium with intensely increased FDG uptake.

**Figure 2:**
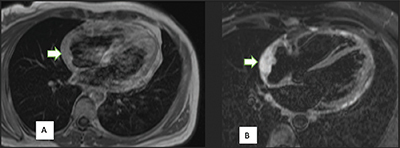
Right atrium angiosarcoma, a broad-based mass in the right atrium associated with thickening of the pericardium, is predominantly isointense to the myocardium on the T1-weighted image (arrow) **(A)**. On the T2-weighted image **(B)**, the mass shows a typical hyperintensity (arrow).

Further investigations have been conducted to determine the cause of the pericardial effusion. Among these investigations, positron emission tomography (PET)-CT (Figure 1) scan revealed a mass in the right atrium with strong fluorodeoxyglucose avidity. Magnetic resonance imaging (MRI) (Figure 2) was performed to provide additional characterization and to assess the relationship of the mass with the surrounding structures. A cardiac biopsy subsequently confirmed the diagnosis of cardiac angiosarcoma.

In our case, the tumor’s proximity to major blood vessels makes the surgical option challenging and associated with a high level of risk. Consequently, the patient underwent chemoradiotherapy, receiving weekly Paclitaxel followed by six cycles of doxorubicin.

**Figure 3:**
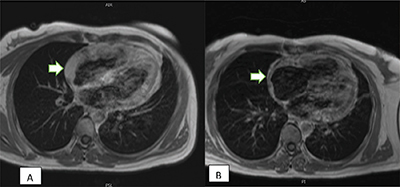
**(A)** Post-contrast T1 imaging before the treatment; **(B) Post-contrast T1 imaging following chemotherapy treatment reveals a partial response with** decreased tumor thickness (arrow).

Subsequent cardiac MRI following chemoradiotherapy showed a significant reduction in the tumor size (Figure 3).

**Figure 4:**
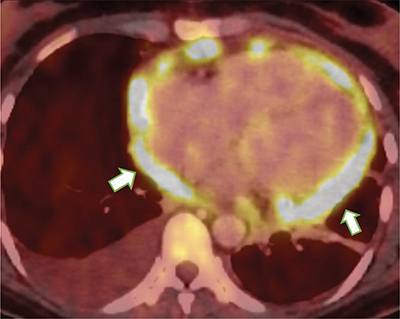
Nodular thickening of the pericardium with intense FDG uptake (arrows).

PET-CT follow-up indicates notable regression in the lesion within the lateral wall of the right atrium, simultaneous with the onset of pericardial carcinomatosis as shown in Figure 4.

## Discussion

Angiosarcoma is an exceptionally uncommon type of soft tissue tumor that originates from the inner lining of blood vessels. Due to its vascular origin, angiosarcoma has the potential to develop in various anatomical locations within the body [4].

However, cardiac angiosarcoma is the most common primary malignant cardiac tumor and represents 33% of the total primary malignant cardiac tumors [5].

Early diagnosis is challenging as patients typically present with nonspecific symptoms, resulting in delayed diagnosis, which, in turn, provides the opportunity for the tumor to invade the surrounding structures such as the aorta and vena cava.

These factors together contribute to the complexity of treatment, as exemplified in our case, where the tumor ultimately becomes unresectable.

In the clinical setting, CT, MRI, and PET-CT are all viable imaging modalities for the evaluation of primary cardiac tumors, with CT and MRI being the primary diagnostic tools.

On CMR T1-weighted images, the mass predominantly exhibits isointensity when compared to the myocardium, occasionally presenting areas of high signal intensity attributable to intralesional hemorrhage. T2-weighted images, on the other hand, often reveal a predominantly heterogeneous and hyperintense appearance [6].

PET-CT can not only be used to differentiate between malignant and benign tumors but also for tumor characterization and staging.

The main treatment for cardiac angiosarcoma involves surgical intervention. Unfortunately, these patients are frequently diagnosed at an advanced stage, rendering surgical intervention challenging and highly risky for the patient.

## Conclusions

Our case confirms the nonspecific clinical presentation of cardiac angiosarcomas and the importance of sectional imaging, notably CT scan, as the first imaging modality in an emergent clinical setting.

Although it is a very rare tumor, radiologists should stay aware of such a possibility upon systematic CT reading, especially if there is pericardial effusion.

## References

[r1] Patel SD, Peterson A, Bartczak A, et al. Primary cardiac angiosarcoma—a review. Med Sci Monit. 2014;20:103–109. DOI: 10.12659/MSM.889875. 24452054 PMC3907509

[r2] Rodriguez Ziccardi M, Tariq MA, Limaiem F, Ahmed SW. Cardiac cancer. Treasure Island: StatPearls Publishing; 2024 January. 2023 January 1. PMID: Bookshelf ID: NBK537144. 30725829

[r3] Randhawa JS, Budd GT, Randhawa M, Ahluwalia M, Jia X, Daw H. Primary cardiac sarcoma: 25-year Cleveland Clinic experience. Am J Clin Oncol. 2016;39:593–599. DOI: 10.1097/COC.0000000000000106. 25036471

[r4] Riles E, Gupta S, Wang DD, Tobin K. Primary cardiac angiosarcoma: A diagnostic challenge in a young man with recurrent pericardial effusions. Exp Clin Cardiol. 2012 Spring;17:39–42. . 23204900 PMC3383367

[r5] Liu C, Zhao Y, Yin Z, et al. Right atrial epithelioid angiosarcoma with multiple pulmonary metastasis confirmed by multimodality imaging-guided pulmonary biopsy: A case report and literature review. Medicine (Baltimore). 2018;97:e11588. 30045289 10.1097/MD.0000000000011588 PMC6078731

[r6] Motwani M, Kidambi A, Herzog BA, Uddin A, Greenwood JP, Plein S. MR imaging of cardiac tumors and masses: A review of methods and clinical applications. Radiology. 2013;268:26–43. DOI: 10.1148/radiol.13121239. 23793590

